# CircRAB3IP upregulates twist family BHLH transcription factor (TWIST1) to promote osteosarcoma progression by sponging miR-580-3p

**DOI:** 10.1080/21655979.2021.1948487

**Published:** 2021-07-05

**Authors:** Guojun Tang, Linghua Liu, Zhihong Xiao, Shuo Wen, Liangyuan Chen, Peng Yang

**Affiliations:** a Department of Spine Surgery, The Second Affiliated Hospital, University of South China, Hengyang, Hunan, P.R. China; b Department of Nursing, Hubei College of Chinese Medicine, Jingzhou, Hubei, P.R. China; c Department of Breast and Thyroid Surgery, Union Hospital West Hospital, Tongji Medical College, Huazhong University of Science and Technology, Wuhan, Hubei, P.R. China; d Department of Breast and Thyroid Surgery, Union Hospital, Tongji Medical College, Huazhong University of Science and Technology, Wuhan, Hubei, P.R. China

**Keywords:** Osteosarcoma, circRAB3IP, miR-580-3p, TWIST1

## Abstract

Circular RNAs (circ RNAs) have been found to play an important role in cancer development. However, the role of circRAB3IP in osteosarcoma (OS) is unclear.

In the present study, We found that circRAB3IP was highly expressed in OS tissues and OS cells. High levels of circRAB3IP was correlated with advanced TNM stage, distant metastasis. CircRAB3IP knockdown inhibited cell proliferation, migration, and invasion. Moreover, circRAB3IP directly binds to miR-580-3p. TWIST1 is directly targeted by miR-580-3p. We also demonstrated that circRAB3IP act as the sponge of miR-580-3p to promote TWIST1 expression. CircRAB3IP promotes OS cells proliferation, migration, and invasion through modulating miR-580-3p/TWIST1 axis. Moreover, circRAB3IP facilitated tumor formation *in vivo*. Our findings suggested that circRAB3IP acts as an oncogene in OS by regulating miR-580-3p/TWIST1 axis.

## Graphical abstract

**Figure:**
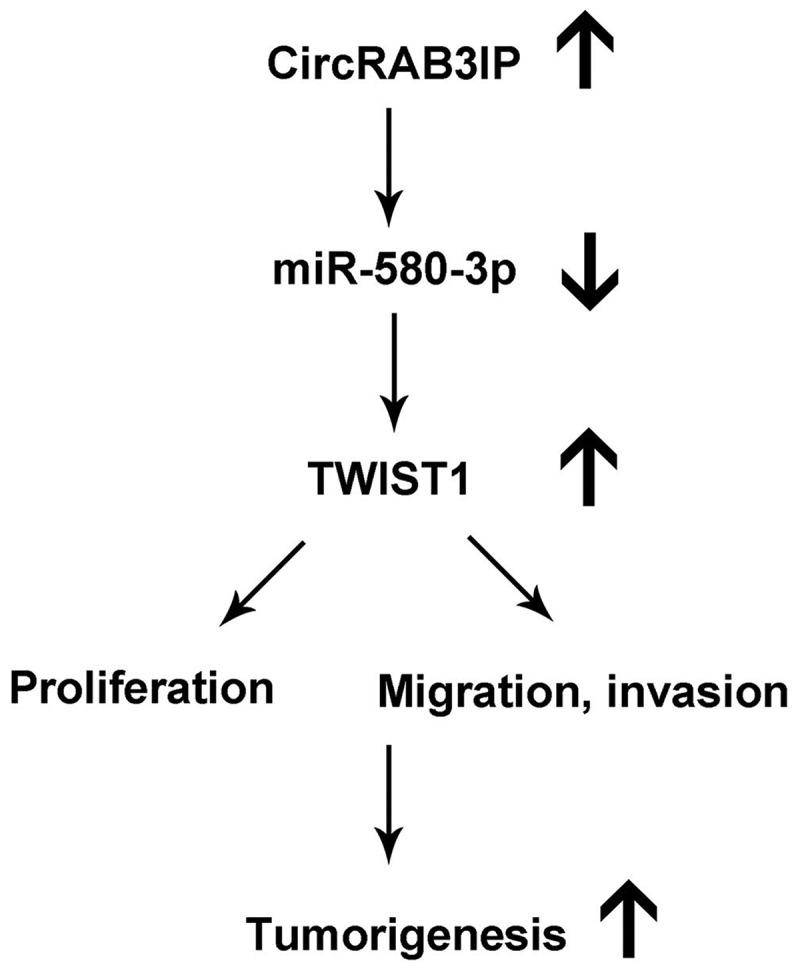

## Introduction

Osteosarcoma (OS) is the most frequent primary malignant bone tumor in the children and adolescents [1]. The exact mechanism of OS initiation and progression has remained unclear. The prognosis of metastatic OS is still poor. Therefore, it is important to explore the molecular mechanism underlying pathogenesis of OS.

Circular RNAs (circRNAs) are a novel subtype of non-coding RNAs (ncRNAs) characterized as covalently closed loop [2]. Studies have been shown that circRNAs participated in many cancer initiations and progressions [3–5]. For example, Fang et al. reported that circMYO10 promoted OS development via regulating miR-370-3p/RUVBL1 [6]. Verduci et al. proved that circPVT1 promotes head and neck squamous cell carcinoma progression through mutant p53/YAP/TEAD transcription-competent complex [7]. Shao et al. showed that circ_0000285 promotes OS progression by sponging miRNA-599 [8]. Although an increasing number of circRNAs play an important role in the tumorigenesis of OS. The regulatory mechanisms of circRNAs in OS pathogenesis need further study. Hsa_circRNA_0000419 (circRAB3IP) named Rab3A-interacting protein (RAB3IP), is located at chr12:70193988–70195501. Previous study reported that circ_0000419 was downregulated in gastric cancer [9]. Zhu et al. showed that circRNA_0000419 was significantly upregulated in OS [10]. However, no further studies have been investigated on circRAB3IP till today.

MicroRNAs (miRNAs) are ncRNAs regulated gene expression and played important roles in the progression of many cancers. For example, Vahabi revealed that miR-96-5p affected radio-chemosensitivity of HNSCC cells by targeting PTEN [11]. In recent years, many studies have reported that circRNA and lncRNA could serve as competing endogenous RNAs (ceRNAs) of miRNAs [6,12,13]. For example, Chen et al. found that LINC00470 bind to miR-580-3p to regulate WEE1 and enhance glioma cell proliferation [14]. Peng et al. showed that LHFPL3-AS1/miR-580-3p/STAT3 axis promotes melanoma progression [15]. Twist1 has been shown to promote osteosarcoma metastasis [16]. However, the mechanistic relationship between miR-580-3p, Twist1 and circRAB3IP in osteosarcoma has yet to be verified.

The aim of our study is to explore the roles of circRAB3IP on the progression of osteosarcoma and reveal the possible mechanisms. We found that circRAB3IP is up-regulated in OS, which is related to the advanced TNM stage and tumor metastasis. We demonstrated that circRAB3IP serves as a sponge for miR-580-3p to up-regulate TWIST1 expression, thereby promoting OS progression. Therefore, our findings proved a novel signaling of circRAB3IP/miR-580-3p/TWIST1 axis involved in OS progression.

## Materials and methods

### Clinical specimens

A total of 42 OS patients at the Second Affiliated Hospital, University of South China, were enrolled in this study. No patient was treated with radiotherapy or chemotherapy before surgery. Adjacent non-tumor tissues and tumor tissues were collected simultaneously. All the resected or biopsy specimens were stored at −80°C. This study was approved by the Ethics Committee of the Second Affiliated Hospital, University of South China. Written informed consents were obtained from all the enrolled patients.

### Cell culture and transfection

The human cell line hFOB1.19 and the human OS cell lines (U2OS,143B, MG63,HOS) were obtained from the Cell Bank of the Chinese Academy of Sciences (Shanghai, China). Cells were cultured in DMEM medium (Invitrogen, Carlsbad, CA, USA) and 10% fetal bovine serum (FBS, Invitrogen).

Short hairpin RNA (shRNA) targeting circRAB3IP, negative control shRNA (sh-NC), circRAB3IP and TWIST1 expression vector, miR-580-3p mimics, mimics NC, miR-580-3p inhibitor, and NC inhibitor were obtained from GenePharma. OS cells were collected, and transferred into a 6-well plate (1.0 × 10^5^ cells/mL), and cultured for 24 h. Then, OS cells were transfected using Lipofectamine® 2000 (Invitrogen, USA) in accordance with the manufacturer’s instruction. Transfection efficiency was detected by quantitative real-time polymerase-chain reaction (qRT-PCR).

### RNA extraction and real-time PCR (qRT-PCR)

Total RNAs were extracted by TRIzol Reagent (Invitrogen). RNA reverse transcription was performed using the Prime Script RT Reagent Kit (TaKaRa, Dalian, China). qPCR was analyzed using the SYBR Green PCR Master Mix Kit (Takara). GAPDH and U6 were used as reference controls. The primer sequences were listed as follows: circRAB3IP (forward: 5′‐TGAGTTGGCTTCAGCTGTTC‐3′, reverse: 5′‐GACACGTCCTGTCCATTGTG‐3′). miR-580-3p (forward: 5′ -GCCGATTGAAGAATGATGAAT-3′, reverse: 5′‐GCAGGGTCCGAGGTATTC‐3′); GAPDH (forward: 5′-CACCCACTCCTCCACCTTTG-3, reverse: 5′-CCACCACCCTGTTGCTGTAG-3′); U6 forward: (5ʹ-CTCGCTTCGGCAGCACA-3ʹ, reverse: 5ʹ-ACGCTTCACGAATTTGC-3ʹ).

### CCK-8 assay

Cell viability was deteced by CCK-8 assay (Dojindo Chemical, Japan). Cells (1 × 103 cells/well) were seeded in 96-well plates and cultured for 0, 24, 48,72 hr. Then, 10 μL

of CCK-8 kit was supplemented to each well. After incubating cells at 37°C for 2 h, the absorbance was measured by spectrophotometrically at 450 nm.

### Colony formation assay

Treated OS cells (1000 cells/well) were cultured in 6-well plates for 14 days. The cell colonies were fixed with formaldehyde for 15 min and stained with 0.5% crystal violet solution for 15 min. The visible colonies were counted under microscope.

### Subcellular fractionation assay

The cytoplasmic and nuclear extracts were extracted from OS cells with NE‐PER Nuclear and Cytoplasmic Extraction Reagents (Thermo Fisher Scientific, Waltham, MA). The distribution of circRAB3IP in cytoplasm or nucleus was analyzed by qRT-PCR analysis. Internal cytoplasmic reference was GAPDH, U6 represented the nuclear RNA control.

### RNA FISH assay

Cy3-labeled circRAB3IP-specific probes were designed and synthesized by RiboBio (Guangzhou, China). The probe signals were detected with a fluorescent in situ hybridization kit (RiboBio) Briefly, when cell confluency reached 70–80%, the OS cells were fixed with paraformaldehyde, and then prehybridized and hybridized in hybridization buffer. Nuclei were stained with DAPI. A fluorescence microscope (Olympus, Japan) was applied to capture the images.

### Transwell assay

Cells (2.0 × 10^4^/well) were seeded in a transwell chamber (Millipore, USA) with a matrigel-coated or without matrigel-coated membrane. The medium containing 10% FBS was added to the lower chamber. The cells were then grown for 24 hours. The cells on the lower surface of the membrane were fixed in 4% paraformaldehyde and stained using 0.5% crystal violet. The stained cells were observed by high-power microscope fields (Nikon, Tokyo, Japan).

### Western blot analysis

Total proteins were prepared using RIPA lysis buffer (Beyotime, China). The Bicinchoninic Acid Protein Assay Kit (Beyotime, China) was used to determine the protein concentration. Equal amounts of protein were separated by 10% SDS-PAGE and electrotransferred onto PVDF membranes (Millipore, USA). Primary rabbit anti‐human antibodies against TWIST1 (1:1000, cell signaling technology, #46702) and GAPDH (1:2000, Abcam, ab9485) were incubated overnight at 4°C. The blots were treated by enhanced chemiluminescence and assessed by One software (Bio-Rad, USA).

### Dual‑luciferase reporter assay

The predicted sequences of circRAB3IP or TWIST1 were inserted into pmirGLO vector (Promega, Madison, WI, USA). OS cells were transfected with miR-580-3p mimics. Then, these cells were co-transfected with vectors carrying circRAB3IP wild type (wt) and mutant type (mut) fragments. Vectors carrying TWIST1 wild type and mutant type fragments were used to transfect OS cells which pre-transfected with miR-580-3p mimics. Forty-eight hours later, the relative luciferase activity was measured using the Dual‐Luciferase Assay System (Promega, Fitchburg, WI, USA).

### Biotin pull‐down assay

Biotin-labeled wild-type miR-580-3p (Bio-wt-miR-580-3p), mutant miR-580-3p (Bio-mut-miR-580-3p) or negative control (NC), synthesized by GenePharma (Shanghai, China) were transfected into OS cells. Forty-eight hours after transfection, OS cells were lysed using lysis buffer, and cell lysate was incubated with M-280 streptavidin magnetic beads (Invitrogen, USA). The bound RNA expressions were detected by RT-PCR.

### Mice xenograft models

The mice (female, BALB/c-nu, 4–6 weeks) were subcutaneously injected with MG63 (2 × 10^6^) cells stably transfected with sh-circRAB3IP or control sh-RNA (sh-NC). Tumor volume was measured every 7 days after implantation. The tumor volume was calculated by the following formula: tumor volume [mm^3^] = (length [mm]) × (width [mm])^2^ × 0.52. Five weeks later, the mice were killed and the tumors were weighed. The animal experiments were approved by the Institutional Animal Care and Use Committee of the Second Affiliated Hospital, University of South China (No. 20200625082, date: 2020.06.25).

### Statistical analysis

SPSS 21.0 software was used for statistics. The data are presented as the mean ± SD of three independent experiments. The one‐way ANOVA or student’s *t* test was used for comparison. *P* < 0 .05 was considered to be significant.

## Results

Previous studies reported that circRAB3IP was highly expressed in gastric cancer and exerted oncogenic functions [9]. However, circRAB3IP expression and its role in OS have not been investigated yet. In this study, we detected circRAB3IP expression in OS, clarified its effects on OS cell proliferation, migration, and invasion and explored the underlying mechanisms.

### CircRAB3IP was upregulated in OS samples and cell lines

**Figure 1.**
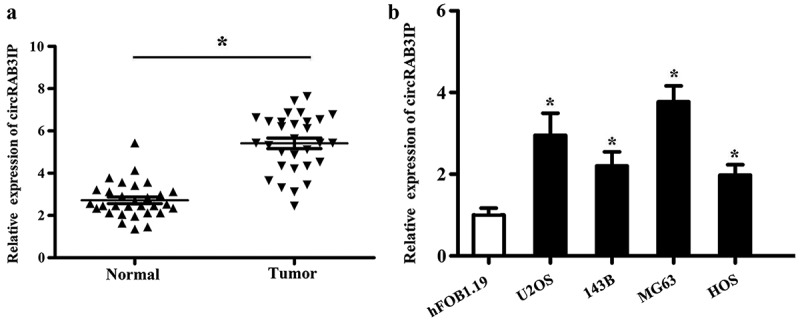
circRAB3IP was upregulated in OS. (a) The expression of circRAB3IP in OS tissues was determined by qPCR. (b) The expression of circRAB3IP in OS cell lines was determined by qPCR. *P < 0.05

We analyzed circRAB3IP in expression by qRT-PCR and found that the expression level of circRAB3IP in OS tissues was remarkably higher than that in adjacent normal tissues (Figure 1a). Meanwhile, qRT-PCR results indicated that circRAB3IP was significantly upregulated in OS cells than in hFOB1.19 cells (Figure 1b). Furthermore, the correlation between circRAB3IP expression and clinicopathological characteristics in 42 OS patients is shown in Table 1. The expression level of circRAB3IP was positively correlated with tumor size, distant metastasis, and advanced tumor stage.

**Table 1.** The correlation between circRAB3IP expression with clinicopathological characteristics of osteosarcoma patients

Characteristics	circRAB3IP	Expression	*p* Value
	Low (***n*** = 21)	**High (*n* = 21)**	
**Age**			
≤18	9	11	0.536
>18	12	10	
**Gender**			
Male	14	12	0.525
Female	7	9	
**Tumor size**			
≤8 cm	8	17	0.004**
>8 cm	13	4	
**Distant metastasis**			
Yes	16	6	0.002**
No	5	15	
**TNM stage**			
I-IIA	15	8	0.029*
IIB-III	6	13	

*p* values were acquired by Pearson’s chi-square test.

*p < 0.05, **p < 0.01 was considered statistically significant.

Low expression of circRAB3IP in 42 patients was defined as a value below the 50th percentile, and high above the 50th percentile.

### CircRAB3IP knockdown inhibited OS cells proliferation, migration, and invasion

**Figure 2.**
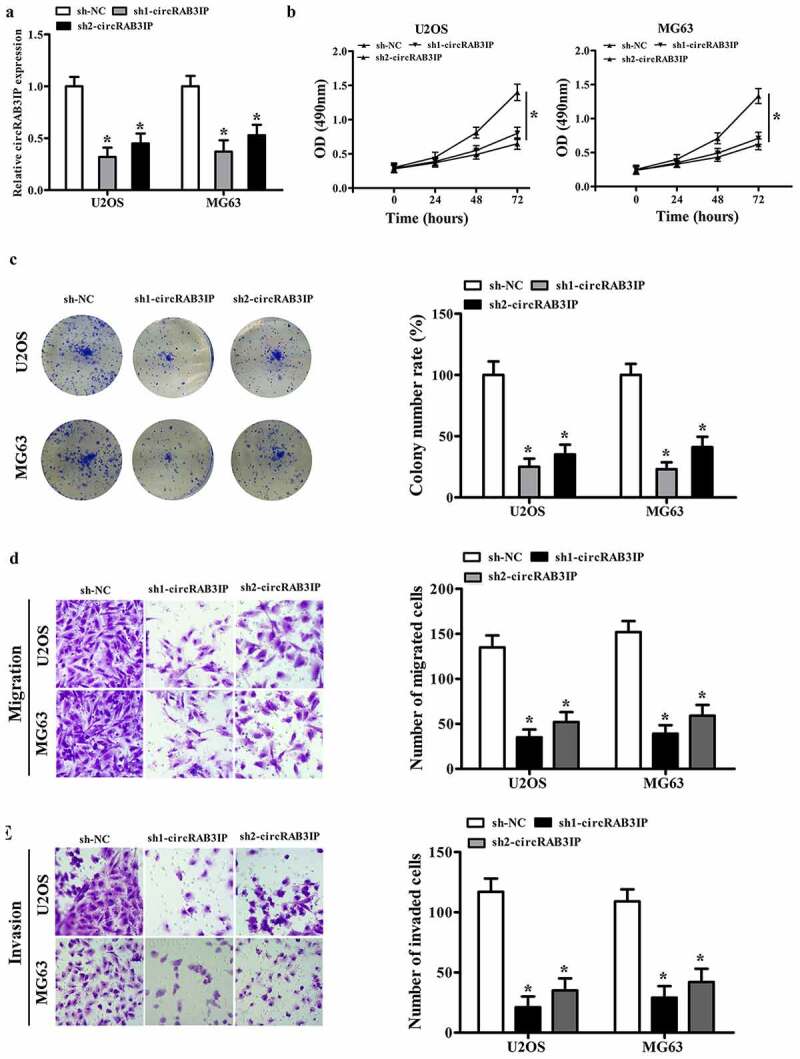
circRAB3IP knockdown inhibited OS cells proliferation, migration, and invasion. (a) circRAB3IP expression was decreased in U2OS and MG63 cells transfected with si-circRAB3IP. (b, c) The CCK-8 assay and colony formation assay were performed to determine the effects of circRAB3IP on cell proliferation of OS cells. (d, e) Transwell assay was used to detect the effects of circRAB3IP on cell migration and invasion of OS cells *P < 0.05

To investigate circRAB3IP biological functions in the progression of OS, circRAB3IP was knocked down in U2OS and MG63 cells using sh-circRAB3IP. sh-circRAB3IP, which targets the junction site of circRAB3IP, significantly inhibited circRAB3IP expression as detected by qRT-PCR (Figure 2a). The CCK8 assay results showed that the viability of U2OS and MG63 cells in sh-circRAB3IP group was reduced than that of the control group (Figure 2b). Furthermore, colony formation assay demonstrated that circRAB3IP knockdown led to a remarkable downregulation of the colony number of U2OS and MG63 cells (Figure 2c). Transwell assay showed that the migration and invasion capacities of U2OS and MG63 cells were inhibited by circRAB3IP knockdown (Figure 2d, e). Taken together, our data indicated that circRAB3IP knockdown suppressed OS cells proliferation, migration, and invasion in vitro.

### CircRAB3IP acted as a sponge for miR-580-3p in OS

**Figure 3.**
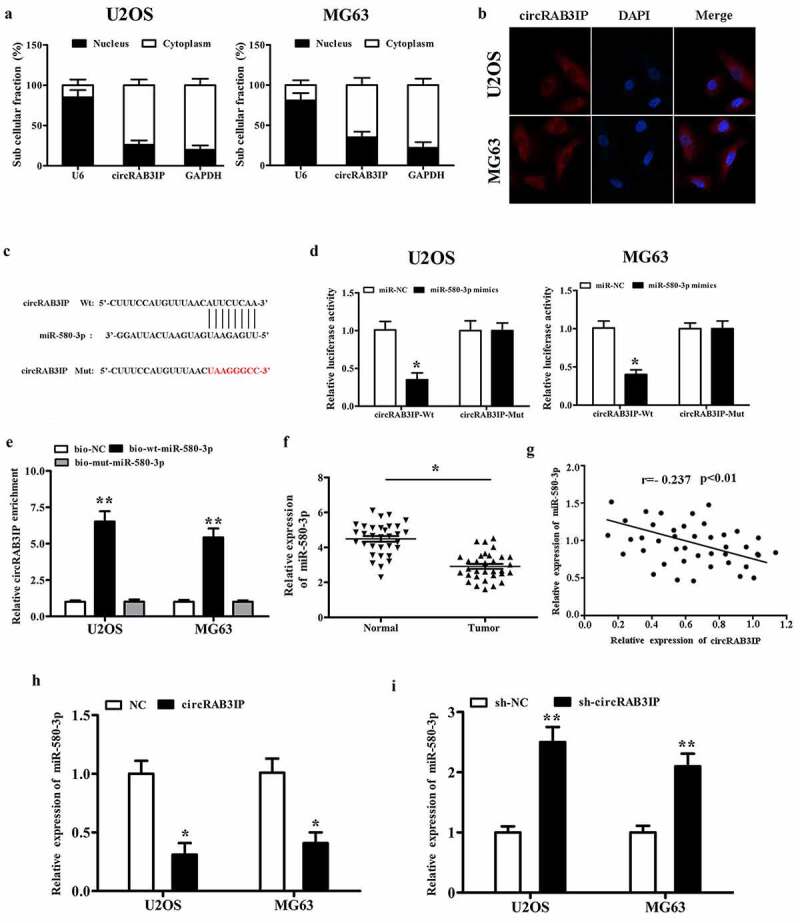
circRAB3IP was a sponge for miR-580-3p in OS cells. (a)The expression of circRAB3IP in cytoplasm. (b) FISH showed that circRAB3IP localized mainly in the cytoplasm. (c) Bioinformatics was used to predict the potential binding site between circRAB3IP and miR-580-3p. (d) Luciferase reporter assay showed the targeting relationship between miR-580-3p and circRAB3IP. (e) Biotin-labeled miR-580-3p (bio-wt-miR-580-3p), bio-mut-miR-580-3p or negative control (bio-NC) were transfected into OS cells, and the streptavidin-captured circRAB3IP was detected by qRT-PCR. (f) qRT-PCR indicated that miR-580-3p was downregulated in OS tissues. (g) Pearson’s analysis showed that circRAB3IP expression was negatively correlated with miR-580-3p expression in OS tissues. (h) circRAB3IP overexpression (oe-circRAB3IP) inhibited miR-580-3p expression. (i) miR-580-3p expression was increased after circRAB3IP knockdown. *P < 0.05, **P < 0.01

CircRNAs have been reported to act as miRNA sponges to regulate gene expression, which plays an important role in many cancer initiations and developments. To investigate the regulatory mechanism of circRAB3IP, we examined the intracellular location of circRAB3IP. Subcellular fractionation and RNA FISH assay indicated that circRAB3IP was predominantly localized in the cytoplasm (Figure 3a, b). To understand the mechanism of circRAB3IP in OS progression, we screened the potential targets of circRAB3IP by Circular RNA Interactome. The Circular RNA Interactome predicted that miR-580-3p had binding sites with circRAB3IP (Figure 3c). To verify the direct interaction of circRAB3IP and miR-580-3p, fragments of wild‐type (wt) and mutated (mut) circRAB3IP sequence containing the putative recognition site of miR-580-3p was cloned into pmirGLO vector. We then co-transfected with both cloned vector and miR-580-3p mimics in OS cells and performed the dual-reporter luciferase assay. Dual-reporter luciferase assay showed that miR-580-3p mimics significantly inhibited the luciferase activity of wild-type circRAB3IP, while did not affect the luciferase activity of circRAB3IP-mut group (Figure 3d). We then performed RNA pull-down assay to validate the interplay between circRAB3IP and miR-580-3p. The results indicated that circRAB3IP could be pulled down by biotinylated miR-580-3p (bio-wt-miR-580-3p) but not by biotinylated negative control (bio-NC) (Figure 3e). We performed qRT-PCR assay to assess the association between circRAB3IP and miR-580-3p. The results showed that miR-580-3p was downregulated in OS tissues (Figure 3f). CircRAB3IP expression was negatively correlated with miR‐580‐3p in OS tissues (Figure 3g). Furthermore, real-time RT-PCR results showed that miR-580-3p expression was remarkably reduced in OS cells after transfected with circRAB3IP overexpressed plasmids and significantly upregulated in OS cells transfected with sh-circRAB3IP (Figure 3h, i). Taken together, our results proved that circRAB3IP acts as a ceRNA through directly sponging of miR-580-3p.

### miR-580-3p targeted TWIST1 in OS

**Figure 4.**
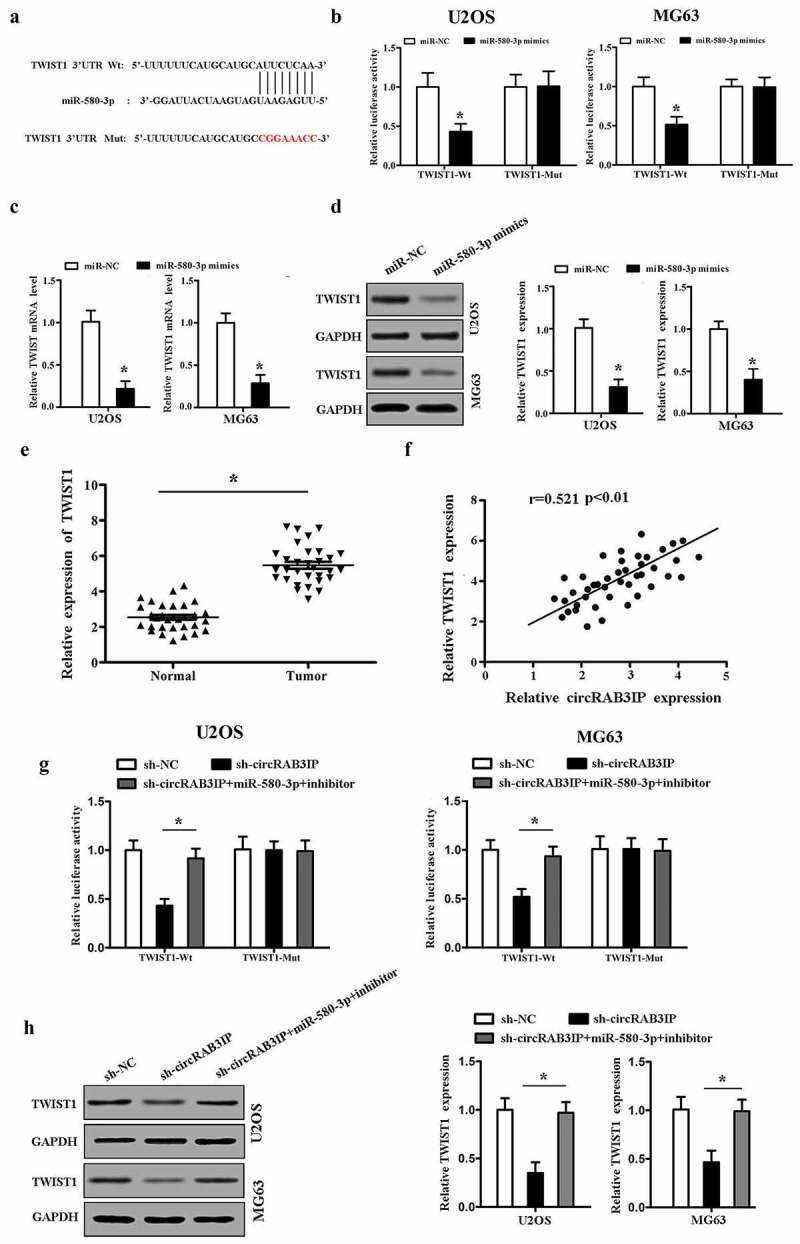
TWIST1 was targeted by miR-580-3p in OS (a) Bioinformatics was used to predict the potential binding site between miR-580-3p and TWIST1. (b) TWIST1 was a target gene of miR-580-3p. (c, d) miR-580-3p mimics inhibited TWIST1 expression. (e) qRT-PCR indicated that TWIST1 was up-regulated in OS tissues. (f) Pearson’s analysis showed that circRAB3IP expression was positively correlated with TWIST1 expression in OS tissues. OS cells were transfected with sh-NC, sh-circRAB3IP, sh-circRAB3IP+miR-580-3p inhibitor. (g) Luciferase reporter assay was used to determined the activity of TWIST1-Wt reporter. (h) TWIST1 expression was detected by Western blot. *P < 0.05

Targetscan (http://www.targetscan.org) predicted that miR-580-3p bind to the 3ʹ-UTR of TWIST1 mRNA (Figure 4a). Then, dual-reporter luciferase assay was performed. The results showed that the activity of wt-TWIST1 reporter was inhibited by miR-580-3p mimics, while the luciferase activity in mut-TWIST1 group showed no observable change (Figure 4b). The dual-reporter luciferase assay result suggested that TWIST1 is a target gene of miR-580-3p. In addition, RT-PCR and western blot results demonstrated that TWIST1 expression was directly inhibited by miR-580-3p in both mRNA and protein levels (Figure 4c, d).

We further explored whether circRAB3IP acted as a sponge of miR-580-3p to promote TWIST1 expression in OS. We found that TWIST1 was upregulated in OS tissues (Figure 4e). TWIST1 expression level in OS tissues was positively correlated with circRAB3IP expression level (Figure 4f). The luciferase reporter activity of wt-TWIST1 was inhibited by sh-circRAB3IP, and the luciferase activity of wt-TWIST1 was rescued by miR-580-3p inhibitor (Figure 4g). Furthermore, circRAB3IP knockdown inhibited the expression of TWIST1, which was reversed by miR-580-3p inhibitor in OS cells (Figure 4h). Our data showed that circRAB3IP regulated TWIST1 expression via sponging miR-580-3p in OS.

### miR-580-3p inhibitor and TWIST1 overexpression reversed the OS cell phenotype induced by circRAB3IP knockdown

**Figure 5.**
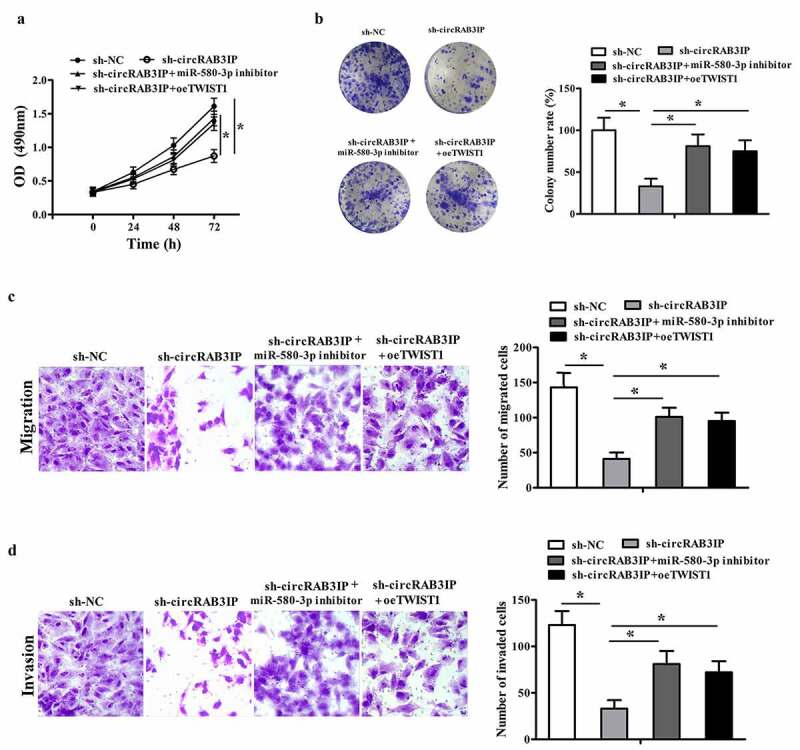
miR-580-3p inhibitor or TWIST1 overexpression reversed effects of sh-circRAB3IP. MG63 cells were transfected with the control sh RNA (sh-NC), sh-circRAB3IP, sh-circRAB3IP+miR-580-3p inhibitor or sh-circRAB3IP+TWIST1 overexpression vector (sh-circRAB3IP+oeTWIST1). (a, b) Cell proliferation was detected by CCK-8 assay and colony formation assay. (c, d) Transwell assay was employed to detect cell migration and invasion of OS cells. *P < 0.05

We further performed rescue experiments to verify the mechanism of circRAB3IP affecting OS cell phenotype. We knockdowned circRAB3IP expression or inhibited miR-580-3p or restored the expression of TWIST1 in MG63 cells. The CCK8 and colony formation assays showed that compared with the sh-circRAB3IP group, the proliferation of MG63 cells was significantly increased in sh-circRAB3IP+miR-580-3p inhibitor group and sh-circRAB3IP+overexpression (oe)-TWIST1 group compare with that in sh-circRAB3IP group (Figure 5a, b). Transwell assays results showed the migration and invasion of MG63 cells in sh-circRAB3IP + miR-580-3p inhibitor group and sh-circRAB3IP+oe-TWIST1 group was significantly increased compared with those in sh-circRAB3IP group (Figure 5c, d).

### CircRAB3IP knockdown inhibited tumor growth in vivo

**Figure 6.**
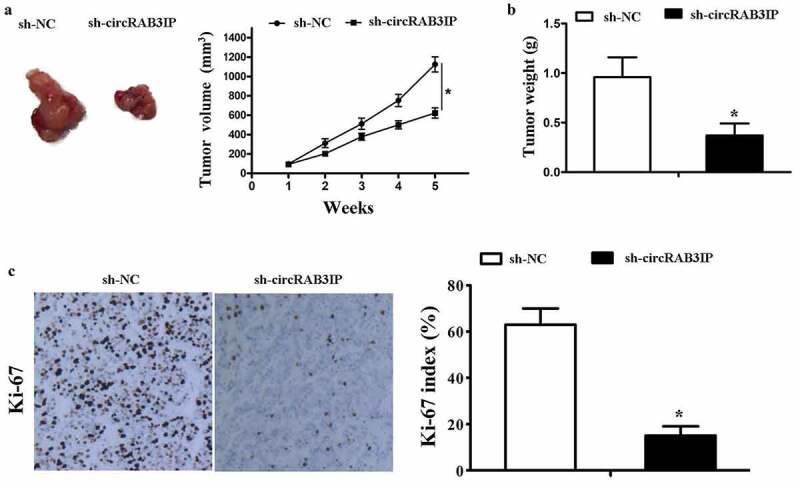
Knockdown of circRAB3IP inhibits tumorigenesis *in vivo*. MG63 cells stably transfected with sh-circRAB3IP or sh-NC were subcutaneously injected into nude mice. (a) After 35 days, tumors were dissected and photographed. Tumor volumes were recorded every 7 days beginning on the day after mice were injected with MG63 cells. (b) Tumor weight was calculated on the day mice were sacrificed. (c) IHC analysis showed a significant decrease in Ki67 expression in the sh-circRAB3IP group compared to that of the control group. *P < 0.05

The above results demonstrated that circRAB3IP was an oncogene in OS which promoted proliferation, migration and invasion of OS cells. To further evaluate the effects of circRAB3IP on tumor growth *in vivo*, we subcutaneously injected MG63 cells which stably transfected with sh-circRAB3IP and control sh-RNA. We found that the tumor volume in sh-circRAB3IP group was significantly smaller than that in control group (Figure 6a). The tumor weight of sh-circRAB3IP group was also smaller than the control group (Figure 6b). The proliferative activity of the tumor cells was assessed by Ki-67, which is a proliferation marker of tumors. The expression of Ki-67 was significantly downregulated in the group of sh-circRAB3IP (Figure 6c). These results showed that circRAB3IP knockdown could markedly suppress OS tumor growth *in vivo*.

## Discussion

Recently, increasing studies have reported the crucial roles of circRNAs in various cancers, especially in the tumorigenesis and progression of OS. For example, CircRNA LRP6 promotes osteosarcoma development by targeting KLF2 and APC [17]. Circ_0000502 facilitates osteosarcoma cell progression by sponging miR-1238 [18]. CircECE1 activates energy metabolism in osteosarcoma through the c-Myc/TXNIP axis [19]. Although circRNAs have been reported to function as biomarkers and play important roles in osteosarcoma, the role and molecular mechanism of circRNAs in OS remain largely unknown. In this study, we focused on the role and underlying mechanism of circRAB3IP in osteosarcoma progression. We found that circRAB3IP is significantly upregulated in OS tissues. High level of circRAB3IP was positively correlated with various clinicopathological parameters. Moreover, we found that circRAB3IP was overexpressed in OS cell lines compared with hFOB1.19 cells, which further indicated potential roles of circRAB3IP in OS. CircRAB3IP knockdown inhibits OS cells growth, migration, and invasion. Furthermore, our results revealed that circRAB3IP promotes OS cell proliferation and metastasis through miR-580-3p/TWIST1 pathway.

It is well known that circRNA/miRNA/mRNA network is the most common regulatory mechanism for circRNAs. CircRNA-miRNA-mRNA axis is reported to play an important role in many cancer progressions, such as tumor cell proliferation, migration, invasion, and apoptosis. For instance, circSLC26A4 promotes cervical cancer progression through regulating miR-1287-5p/HOXA7 [20]. Circ_001621 facilitates osteosarcoma progression by sponging miR-578 [21]. In the present study, subcellular fractionation assay and RNA FISH showed circRAB3IP was mainly distributed in cytoplasm of OS cells; therefore, we hypothesized that circRAB3IP might serve as a competitive endogenous RNA in OS.

A previous study has revealed that miR-580-3p was sponged by lncRNA LHFPL3-AS1 and involved in the progression of melanoma [22]. Another study reported that circ_0072309 inhibited NSCLC progression by sponging miR-580-3p [23]. These studies suggested that miR-580-3p can be sponged by different circRNAs or lncRNAs and exerts various biological functions in different tumors. In this study, our data showed that miR-580-3p was downregulated in osteosarcoma tissues. Furthermore, miR-580-3p expression was negatively regulated by circRAB3IP in OS cells, suggesting that circRAB3IP acts as miR-580-3p sponge. We found that miR-580-3p directly targets TWIST1. Moreover, we found sh-circRAB3IP induced inhibition of cell proliferation, migration, and invasion could be reversed by miR-580-3p inhibitor.

TWIST1 is a transcription factor that induce epithelial–mesenchymal transition (EMT), cell migration, and invasion [24,25]. TWIST1 is overexpressed in various cancer cells including osteosarcoma cancer cells [26]. It has been reported that TWIST1 signaling is involved in lncRNA AFAP1-AS1-induced osteosarcoma tumorigenesis and EMT [27]. A recent study proved that lncRNA JPX promotes lung cancer progression via modulating miR-33a-5p/TWIST1 axis [28]. Our study found that TWIST1 acted as a target of miR-580-3p and was coordinately upregulated with circRAB3IP in OS. Restored TWIST1 expression partially rescued circRAB3IP knockdown-induced inhibition of cell proliferation, migration, and invasion. Widely reported as a transcription factor and an oncogene, TWIST1 promots many cancer metastases and progressions by regulating multiple genes. Studies reported that TWIST1 increased the expression of vimentin, AKT2, Bmi1, and Wnt5a to promote EMT, cell migration, invasion, and metastasis [29–31]. In our study, we revealed that TWIST1 was regulated by circRAB3IP/miR-580-3p, and the downstream genes of circRAB3IP/miRNA-580-3p/TWIST1 need to be further investigated.

## Conclusions

In summary, our study showed that the circRAB3IP/miRNA-580-3p/Twist1 axis act as a novel pathway, regulating OS progression. Our findings suggested that circRAB3IP may serve as a potential therapeutic target for the OS treatment.

## Data Availability

The data used to support the findings of this study are available from the corresponding author upon request.

## Highlights

(1) CircRAB3IP was upregulated in osteosarcoma.

(2) CircRAB3IP promoted osteosarcoma cell progression.

(3) CircRAB3IP promoted osteosarcoma progression through miR-580-3p/TWIST1 axis.
